# Using change trajectories to study the impacts of multi-annual habitat loss on fledgling production in an old forest specialist bird

**DOI:** 10.1038/s41598-017-02072-w

**Published:** 2017-05-12

**Authors:** Eric Le Tortorec, Niina Käyhkö, Harri Hakkarainen, Petri Suorsa, Esa Huhta, Samuli Helle

**Affiliations:** 1 Department of Biological and Environmental Science, University of Jyväskylä, P.O. Box 35, FI-40014 Jyväskylä, Finland; 2 Department of Biology, University of Turku, FI-20014 Turku, Finland; 3 Department of Geography and Geology, University of Turku, FI-20014 Turku, Finland; 4 Natural Resources Institute Finland, Rovaniemi Research Unit, P.O. Box 16, FI-96301 Rovaniemi, Finland

## Abstract

The loss and subdivision of habitat into smaller and more spatially isolated units due to human actions has been shown to adversely affect species worldwide. We examined how changes in old forest cover during eight years were associated with the cumulative number of fledged offspring at the end of study period in Eurasian treecreepers (*Certhia familiaris*) in Central Finland. We were specifically interested in whether the initial level of old forest cover moderated this relation. We applied a flexible and powerful approach, latent growth curve modelling in a structural equation modeling (SEM) framework, to create trajectories describing changes in old forest cover through time, and studied how this change at both the territory core and landscape scales impacted fledging numbers. Our main finding was that at the territory core scale the negative impact of habitat loss on fledging numbers was lessened by the higher levels of initial forest cover, while no association was found at the landscape scale. Our study highlights a powerful, but currently under-utilised methodology among ecologists that can provide important information about biological responses to changes in the environment, providing a mechanistic way to study how land cover dynamics can affect species responses.

## Introduction

The loss and subdivision of habitat into smaller and more spatially isolated units (i.e. habitat loss and fragmentation) due to human actions have been shown to adversely affect species worldwide. A vast amount of research studying the effects of modified landscapes on species has been conducted using individual static snapshots of habitat data combined with biological data^1, 2^. These studies have greatly advanced our understanding of how species respond to habitat structure at different spatial scales. However, the temporal frequency of biological data has often been much higher than that of habitat data, i.e. few landscape data points in relation to the biological data points, which has limited the potential to make temporal inferences of the impacts of landscape change on species. For example, many studies have been unable to model temporal patterns of habitat change at temporal frequencies relevant to the biological question at hand, missing potentially important dynamics^3^, or have been unable to reliably distinguish random noise in time series, caused by e.g. classification errors, from true change^4^.

As access to free regional and global spatial data sets has substantially increased^5, 6^, recent years have witnessed a growing number of studies quantifying spatially and temporally explicit habitat change in various ecosystems^7–9^. A number of empirical studies have also incorporated temporal aspects into studies of habitat loss and fragmentation. These studies have shown clear negative effects of habitat loss and fragmentation occurring through time on organisms, being associated with decreased numbers of individuals and increased local extinctions^10^, decreased persistence and occupancy of individuals within landscapes^11^ as well as decreased species richness and abundance^12, 13^. However, a common shortcoming shared by these studies is that the temporal frequency of habitat data has often been sparse. For example, a study that spans a time period relevant to the biological question at hand might include only one or two time points, which might lead to the study missing potential dynamics taking place between the time points. In addition, previous studies have used the estimates of change in subsequent separate analyses to explore species responses, as opposed to conducting the entire analysis rigorously in one statistical framework taking the statistical uncertainty related to change estimation appropriately into account.

A more robust method of studying environmental changes is to use spatio-temporal change trajectories, which can quantify changes in environmental variables by considering multiple years of data simultaneously. This makes them a much more effective method of characterising change than bi-temporal approaches, where only the difference between the beginning and end states are considered^14, 15^. Compared to bi-temporal approaches, trajectory-based methods are more reliable to quantify rates and dynamics of changes^16^, less sensitive to seasonal and year-to-year variation^4^ as well as to potential land cover classification errors present in landscape data. By identifying changes in environmental variables through time, trajectory-based methods lessen the impact of outliers and missing data points. A statistical framework combining change trajectories of environmental data through time with biological data in a single statistical framework would thus be highly informative when linking environmental change to species responses.

**Figure 1:**
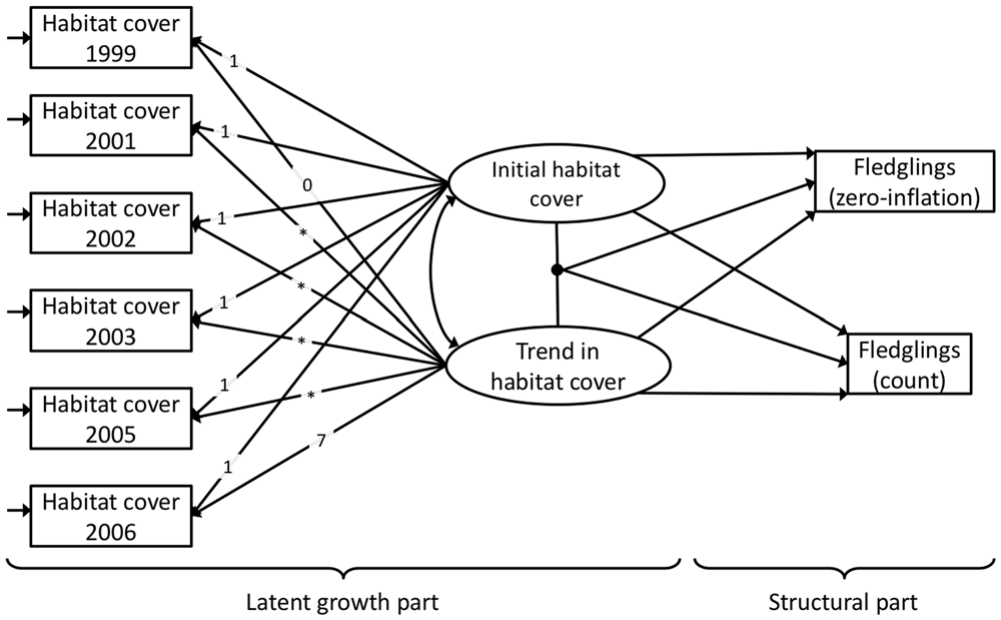
Graphical representation of baseline latent growth curve models used to examine the influence of change in habitat cover, initial habitat cover and their interaction on the cumulative number of fledged offspring at the end of the study period. The boxes on the left represent observed habitat data collected from individual years from which initial habitat cover and change in habitat cover, as well as their interaction are derived from. Single-headed arrows in the latent growth curve part of the model (on the left side) represent factor loadings, connecting the observed annual habitat cover and unobserved latent intercept and slope factors. Numbers along these arrows mean fixed values, while asterisks denote estimated values from the data. Single-headed arrows in the structural part of the model (on the right side) represent structural path coefficients examining how intercept and slope factors and their interaction influence cumulative fledgling number. The arrows originating from the connecting dot between initial habitat cover and change in habitat cover represent latent interactions between the latent factors. Covariance between initial habitat cover and change in habitat cover is depicted as a double-headed arrow. Short arrows pointing at the habitat variables represent their residual variances. Please note that these baseline models excluded residual covariances between habitat cover that we included in final models.

We studied how the number of fledglings in the Eurasian treecreeper (*Certhia familiaris*), hereafter the treecreeper, in Central Finland was associated with changes in habitat cover in the immediate vicinity of the nest box (territory core scale) and the surrounding landscape (landscape scale) over a period of eight years. We summed the number of fledged offspring per nest box site over the entire study period, which enabled us to quantify the cumulative impacts of continuing habitat change on species that could have been easily missed when using static snapshots of habitat data. We used percent cover of forest over 50 years in age as an estimate of habitat amount since it has previously been shown to be associated with territory occupancy^17^ and nestling body condition of treecreepers^18^ at the territory core scale, and nest predation^19, 20^ at the landscape scale. Importantly, we also investigated whether the level of initial habitat cover moderated the influence of change in habitat cover on treecreeper fledging numbers. The data was analysed using latent growth curve modelling in a structural equation modeling (SEM) framework^21^ (Fig. 1), which is a flexible and powerful multivariate approach, yet still virtually unknown to ecologists^22^. For both scales, separately, we decomposed the data on annual amount of habitat cover into initial level of habitat at beginning of the study period and into change in habitat cover during the study period. These were then related to the number of fledged offspring and the excess of zero fledglings (a zero- inflated model was used due to a predominance of zeros), which enabled us to simultaneously model habitat change and its association on the cumulative number of fledglings per nest box site in a single statistical model.

Based on our previous results on this system^17, 20^, we hypothesised that: (1) decreasing habitat cover at the territory core scale would decrease the number of fledged offspring by reducing the total number of nesting events, (2) decreasing habitat cover at the landscape scale would increase the number of fledged offspring because of lower predation pressure, and (3) a high level of initial habitat cover in the nesting site would protect the sites against the negative influence of decreasing habitat cover.

## Results

**Figure 2:**
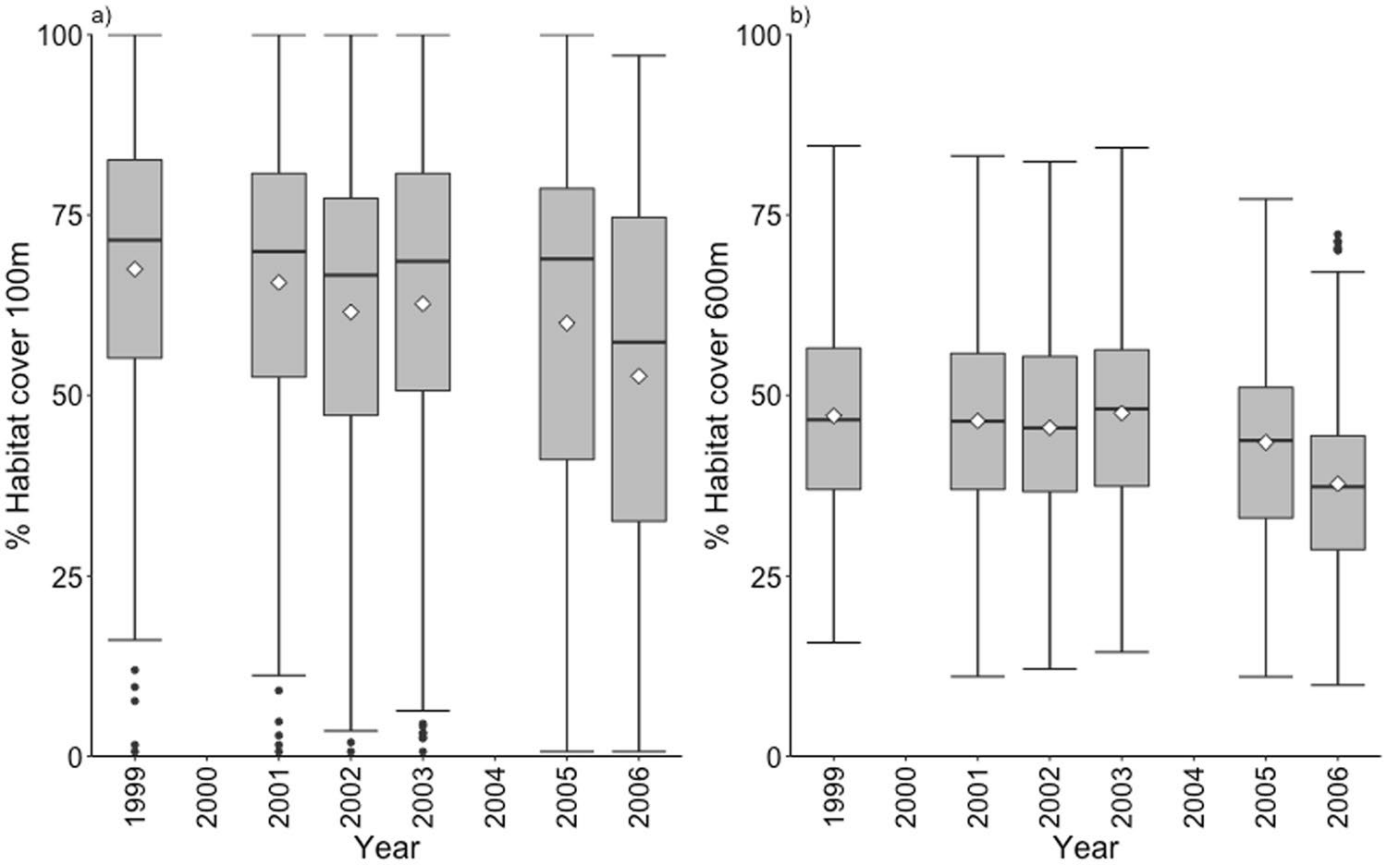
Box plots showing change in habitat cover at the territory core (100 m) (**a**) and landscape (600 m) (**b**) scales during the study period of eight years. The box plots show raw data of percent cover of habitat (old forest) of nest box sites for each year separately. The tops and bottoms of the grey boxes represent the 75^th^ and 25^th^ percentiles, respectively, while the lines in the middle of the grey boxes represent the median and the white diamonds show the mean. The whiskers above and below the grey boxes represent 1.5 times the inter-quartile range, and the individual points represent values beyond these.

At the beginning of the study period, nest box sites had an average (SD) of 66.8% (20.9) and 47.7% (14.8) of habitat cover at the territory core and landscape scales, respectively (Fig. 2). The part of the latent growth curve model describing change in habitat cover in these nest box sites indicated that, as expected from the study design, initial habitat cover varied significantly between the nest box sites at both scales. This was evident by the significant variances for the corresponding latent intercepts at both scales (Table 1). There was also a statistically significant average decrease in habitat cover at both spatial scales. Habitat was lost on average at the rate of 2.2 and 1.4% per year at the territory core and landscape scales, respectively (Table 1, Fig. 2). We also found variation from this average change between nest box sites, suggesting that the rate of habitat loss varied between nest box sites (although at the landscape scale the significance of variance for change in habitat cover was marginal; Table 1). The covariance between the initial level of habitat cover and change in habitat cover was significant for both scales: at the territory core scale, the nest box sites with high levels of initial habitat cover lost less habitat than those with less initial habitat, while this relation was in opposite direction at the landscape scale (Table 1).

**Table 1:** Results of two separate latent growth curve models examining how change in habitat cover, initial level of habitat cover and their interaction at the territory core (100 m) and the landscape (600 m) scales were associated with the total number of fledged offspring produced during the study period.

	100 m			600 m		
	Estimate	SE	P-value	Estimate	SE	P-value
Structural path coefficients						
*Negative binomial count model*						
Initial habitat cover	0.02	0.07	0.776	−0.011	0.079	0.895
Change in habitat cover	2.886	1.31	0.060	0.536	2.326	0.818
Initial cover × Change	−0.361	0.195	0.064	0.234	0.543	0.666
*Zero-inflated logistic model*						
Initial habitat cover	−1.75	1.187	0.140	−0.301	0.391	0.441
Change in habitat cover	33.558	28.205	0.234	−4.755	11.152	0.818
Initial cover × Change	−4.21	3.797	0.268	1.437	2.688	0.593
Latent variable means						
Initial habitat cover	6.775	0.149	<0.001	4.806	0.103	<0.0001
Change in habitat cover	−0.22	0.022	<0.001	−0.142	0.008	<0.0001
Latent variable variances						
Initial habitat cover	3.205	0.491	<0.001*	1.998	0.184	<0.0001*
Change in habitat cover	0.033	0.014	0.001*	0.005	0.003	0.001*
Basis coefficients						
1999	0	Fixed	—	0	Fixed	—
2001	0.762	0.438	0.082	1.067	0.237	<0.001
2002	2.835	0.605	<0.001	1.832	0.249	<0.001
2003	2.834	0.667	<0.001	0.503	0.275	0.068
2005	4.315	0.488	<0.001	3.029	0.231	<0.001
2006	7	Fixed	—	7	Fixed	—
Latent variable covariances						
Initial habitat cover – Change in habitat cover	0.121	0.038	0.001*	−0.033	0.011	<0.001*
Residual covariances						
Years 1999, 2001	0.68	0.241	0.005	0.048	0.022	0.029
Years 2001, 2002	0.387	0.143	0.007	0.016	0.01	0.122
Years 2002, 2003	0.698	0.214	0.001	0.014	0.011	0.180
Years 2003, 2005	0.339	0.121	0.005	0.054	0.015	<0.0001
Years 2005, 2006	0.59	0.24	0.014	0.041	0.039	0.295
Residual variances						
Year 1999	1.37	0.187	<0.0001	0.242	0.047	<0.0001
Year 2001	1.37	0.187	<0.0001	0.092	0.018	<0.0001
Year 2002	1.37	0.187	<0.0001	0.103	0.02	<0.0001
Year 2003	1.37	0.187	<0.0001	0.07	0.02	0.001
Year 2005	1.37	0.187	<0.0001	0.124	0.02	<0.0001
Year 2006	1.37	0.187	<0.0001	0.144	0.098	0.141

Note that regression coefficients on negative binomial and zero-inflated logistic models are on log and logit scales, respectively, and that the p-values of factor variances are halved because, by definition, these cannot be negative (Hox 2010).

*Statistical significance determined with likelihood ratio test.

**Figure 3:**
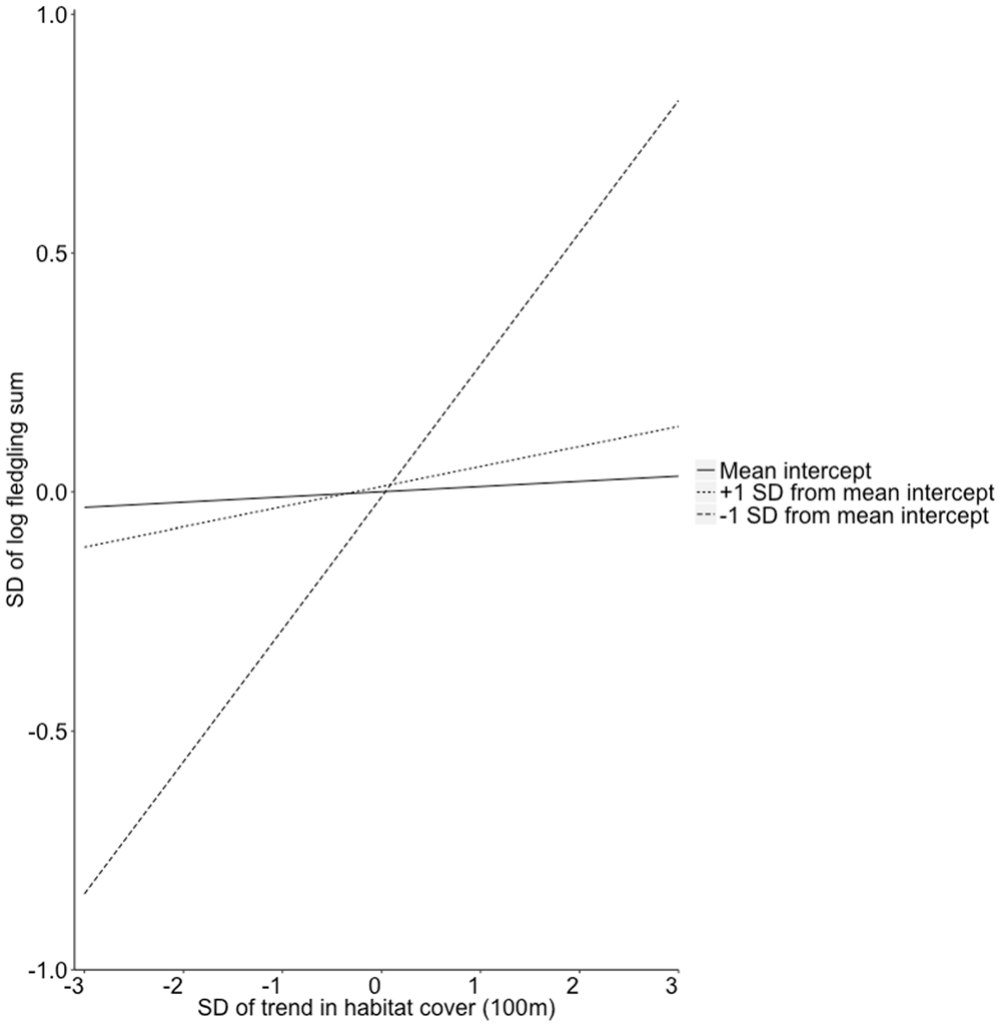
Graphical representation of the moderating influence of the initial level of habitat cover in a nest box site on the association between change in habitat cover and the total number of fledged offspring at the end of the study period at the territory core scale. The solid line represents the response, in standard deviations, of the sum of fledged offspring in response to a change in habitat cover. The dotted line represents the same association for nest box sites with initial habitat cover one standard deviation above the average, while the dashed line represents nest box sites with initial habitat cover one standard deviation below the mean. In nest box sites where initial habitat cover was below the mean level, treecreeper fledging number was more strongly impacted by habitat change than in sites where initial habitat cover was at or above the mean level.

**Figure 4:**
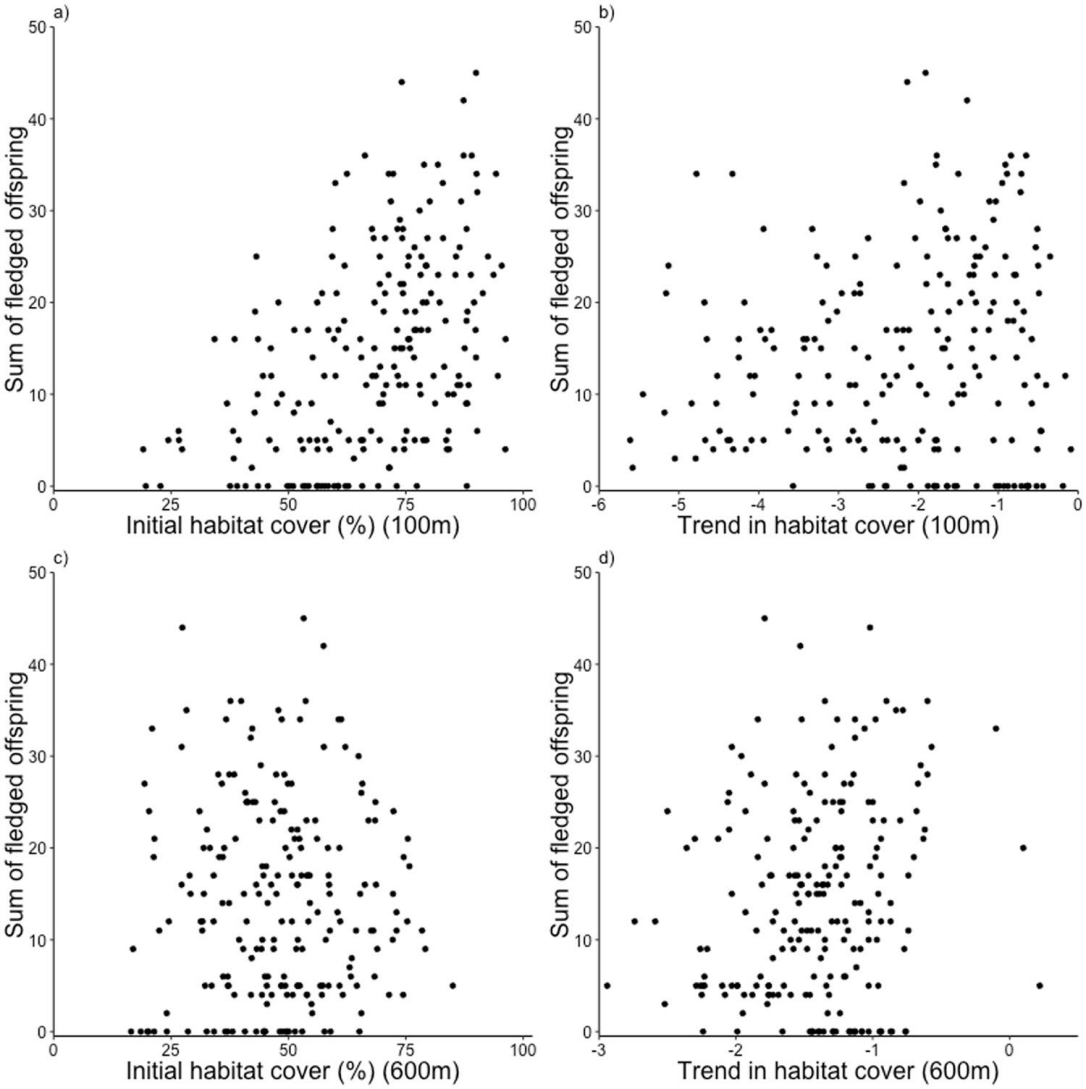
Scatter plots showing the association between the level of initial habitat cover and the number of fledged offspring summed per nest box site, and the association between change in habitat cover, measured by factor scores, and the summed number of fledged offspring. Panel (a) shows the association between the initial level of habitat cover and the number of fledged offspring at the territory core scale, while panel (b) shows the association between change in habitat cover and fledged offspring at the same spatial scale. Panels (c) and (d) show the same associations at the landscape scale. Note that factor scores are set on an arbitrary scale, with the mean value corresponding to the value of the factor mean (see Table 1).

When associating changes in habitat cover with the cumulative number of treecreeper fledglings at the end of the study period, we found that at the territory core scale, initial habitat cover, change in habitat cover and their interaction did not predict the excess of zero fledglings (sites that produced no fledged offspring) (zero-inflated logistic model in Table 1, Fig. 1). Instead, we found a statistically significant interaction between change in habitat cover and initial habitat cover predicting the number of fledglings (negative binomial model in Table 1). That is, increasing habitat loss during the study period, which decreased the cumulative number of fledglings, was attenuated in nest box sites with high initial habitat cover. When considering variance-standardised results, in nest box sites with initial habitat cover one standard deviation above the mean, a decrease of one standard deviation in habitat change (i.e. more habitat loss) decreased the expected number of fledglings by 4.3% (Fig. 3). In nest box sites with initial habitat cover one standard deviation below the mean, a one standard deviation decrease in habitat change decreased the expected number of fledglings by 32.3% (Fig. 3). Initial habitat cover, change in habitat cover or their interaction were not associated with the excess of zero fledglings nor the number of fledged offspring at the landscape scale (Table 1, Fig. 4).

## Discussion

A number of previous studies have shown that habitat loss, quantified from snapshots of habitat data, influence important traits associated with individual reproductive success, such as nest site occupancy, clutch size and the number of fledged offspring^17, 23, 24^. This study complements this literature by explicitly associating multi-annual changes in habitat cover with nest- site level production of offspring in a single statistical framework. The treecreeper nesting sites in our study area experienced a clear decrease in the cover of old forest, which is the main habitat for these birds. On average, nest box sites lost roughly 17% habitat cover at both spatial scales during our study period of eight years. Taking advantage of a latent growth curve modelling in a structural equation modeling framework we found that at the territory core scale the association between rate of habitat loss and the number of fledged treecreeper young seemed to be moderated by the amount of initial habitat cover: the harmful influence of habitat loss on fledgling numbers was reduced in nest box sites with high initial habitat cover compared with those with less initial habitat cover. At the landscape scale, we did not find any influence of temporal habitat loss on fledging numbers.

We found support for the protective effect of high levels of initial habitat cover against the negative impact of temporal habitat loss on fledging numbers is treecreepers. This effect seemed rather strong as in nest box sites with initial habitat cover one standard deviation below the mean, a one standard deviation decrease in habitat cover over time decreased the expected number of fledglings by 32.3%. This finding highlights the importance of considering multiple habitat characteristics simultaneously in studies of habitat-related influences on species responses. Previous studies in our study population, using static snapshots of habitat structure, have also shown a negative influence of habitat loss on fine-tuned responses such as physiological stress in nestlings^18, 19^. Our approach of characterising changes in habitat cover combined with aggregated biological data enabled us to quantify subtle influences of continuing habitat change on species that could have been easily missed when using static snapshots of habitat data. The influence of change in habitat cover on fledging numbers at the territory core scale is in line with the results of Suorsa *et al*.^17^, where the cover of old forest immediately surrounding the nest site had a strong positive association with territory occupancy. In a previous study we did not find any evidence that high quality individuals occupied the largest forest patches^25^, suggesting that the results seen here are not likely due to the highest quality individuals breeding in sites with the most old forest. Interestingly, we did not find evidence that changes in habitat cover or initial habitat cover at the landscape scale had any impact on fledging numbers. This suggests that the main mechanism impacting the long-term number of fledglings in this species was nest occupancy and resource availability, which are mainly determined by habitat variables surrounding the nest^17^, rather than nest predation, which is influenced by habitat variables at the landscape scale^19^.

Despite the robust statistical methods used in this study, there are some potential sources of error that may have influenced our results. First, it is possible that the definition of habitat used in this study was too broad to show the full influence of change in habitat cover on treecreepers. Suorsa *et al*.^17^ showed clear area-sensitivity in the occupancy of treecreeper nest box sites, but the wood volume used in that study was over 150 m^3^/ha, which was higher than the level used in this study (100 m^3^/ha). However, several previous studies have shown that the same habitat definition used in this study can affect both physiological and life history traits in treecreepers^18–20^. Second, errors in the classification of satellite images might have resulted in false change in the results, for example showing decreases in habitat cover in sites that had stable habitat cover. However, a previous classification accuracy assessment^20^ of the latest image used in this study showed that classification errors should not have been a serious problem here. In addition, our method of fitting a trend through the time series for each nest box site lessened the impact of potential classification errors. Third, we did not take the potential impact of weather on treecreeper fledging numbers into account due to sample size limitations in the statistical models. Although weather conditions during the nesting season can have strong impacts on reproductive success in birds^26^ we did not find any impact of temperature or rain during the nesting season on treecreeper reproductive success in a previous study^20^, suggesting that its omission unlikely produced serious bias to our results. Finally, owing to our moderate sample size (i.e. the number of nest box sites), our analysis might have suffered from inadequate statistical power to detect subtle effects of habitat loss on treecreeper fledging numbers. General rules regarding the sample sizes needed are hard to come by in latent growth curve literature and depend e.g. on model complexity^21^. In addition, a lack of *a priori* knowledge of the expected effects sizes means that *post hoc* power analyses are not suitable.

Our study shows the utility of a powerful modelling approach by demonstrating the applicability of latent growth curve modelling in simultaneously describing environmental change and relating this to an aspect of reproductive success. Latent growth curve modelling is akin to multilevel (or hierarchical or mixed) modelling that most ecologists are familiar with. However, it provides a more flexible framework to model complex multivariate longitudinal processes than a multilevel framework when the number of levels is limited to three or less^21^. In addition, the interaction between initial habitat cover and subsequent change in habitat cover on treecreeper fledging numbers was straightforward to model in a SEM framework, but would have been cumbersome to do in standard multilevel framework^27^. Instead of studying here how changes in habitat cover influence fledging numbers of the species we could have also included time-variant and time–invariant predictors (e.g. climatic or policy decisions) directly influencing environmental change *per se* as well as species outcome(s). Inclusion of measurement error in variables of interest by using common factors is also readily implemented in a latent growth curve approach, which may result in less causally inconsistent estimates and increased statistical power^28^. As a downside, complex models need generally more data, which may hinder SEM’s applicability in small data sets. We however expect this to be no obstacle in the upcoming era of big data.

Since roughly 85% of the forested area in Finland is not protected^29^, the ecologically sustainable management of forests subjected to commercial activities is critical for the overall retention of biodiversity in the country. As such, determining the effects of rates of change on biological responses provides important information that can be used to plan long-term management of forest stands. In terms of forest management, the results of this study support the creation of fewer large areas of habitat that can protect species against changes in habitat cover, versus preserving multiple small patches where the negative effects of habitat loss will be magnified, leading to local extinctions and smaller population capacity^30^. However, it is important to keep in mind that this study was conducted on a single species, and as such these results might be species-specific. We suggest that studies using a similar change detection methodology but inspecting population- and community- level responses in different landscapes could yield management recommendations that are applicable in broader contexts. In addition, the methodology outlined here offers a way to inspect potential threshold^31^ rates of habitat change after which its effects clearly increase.

## Materials and Methods

### Treecreeper data

The treecreeper is a small area-sensitive passerine, which prefers old forests (>50 years old) as breeding habitat^17^. It feeds by searching for invertebrates on tree trunks^32^ and constructs a nest under a flap of loose bark or in crevices in tree trunks^33^, but also readily accept specially designed nest boxes. The data used in this study were collected between 1999 and 2006 from a study site covering 1150 km^2^ in Central Finland (centred on 62°37′N, 26°20′E). During the study period this area was subject to intensive commercial forestry whereby forests are clear cut, replanted and thinned in a roughly 80-year cycle.

The study area consisted of a total of 241 nest box sites selected to include fragmented, intermediate and unfragmented landscapes. Each site contained two identically sized nest boxes placed 30 m apart to facilitate potential second breeding attempts within the breeding season. Although some individual treecreepers nested in natural cavities in our study site, these numbers were so low (0–3 per year for the entire study site) that their impact on the study must have been negligible. Throughout the breeding season (April – July) each nest box site was visited at least two times to identify occupied sites. Occupied sites were further visited throughout the breeding season to determine first and second breeding attempts, count the number of eggs laid, ring and measure nestlings and capture and measure the parents. Predation events could be identified on the state of the nest, and the final number of fledged offspring was determined by subtracting the number of deceased chicks from the number of nestlings. More detailed information of the study protocols can be found in Huhta *et al*.^19^. For the current study, the number of fledglings was not summed per individual female treecreeper because these only nested on average (SD) 1.3 (0.62) times in our study population. Instead, the number of fledglings was summed per each nest box site for the whole study period. This was done to maximise the number of nest box sites included in the study. Despite the high turnover of individuals breeding in a single nest box site we did not find any evidence that more experienced individuals or those in better condition occupied sites with the most old forest^20^. In order to assess change in habitat cover we used only nest box sites from which at least two consecutive years of landscape data was available from the same location (i.e. the nest box site had not been moved), which resulted in 213 nest box sites. These nest box sites had an average (SD) of 5.3 (0.96) years of habitat data (out of six separate habitat data points). From the nesting data, we excluded nesting events where experimental procedures for previous studies had taken place during the study duration (n = 149), which resulted in a total of 1009 separate nesting events by 475 individual female treecreepers. Each nesting site produced an average of 14.4 (10.6) fledged offspring during the entire study period. Nest box sites had an average of 7.78 (0.57) years of nesting data: two sites had four years of data, four sites had six years, 30 sites had seven years, and 177 had no missing years.

All methods were carried out in accordance with relevant guidelines and regulations, and our study complies with the current laws of Finland. All experimental protocols were approved by the Environmental Centre of Central Finland.

### Quantifying changes in habitat cover

Habitat change data were generated from Landsat 5 Thematic Mapper satellite images (resolution 30 m × 30 m) for the years 1999, 2001, 2002, 2003, 2005 and 2006 (2000 and 2004 were missing due to lack of cloud-free images), downloaded from the United States Geological Survey Global Visualization Viewer service (http://glovis.usgs.gov). The entire study area was covered with one image, and for each year we selected one image with the minimum amount of cloud cover, taken between the first of May and last of September. For 2006 a cloud-free composite of two sequential cloudy images was used. Each satellite image was classified into two classes with supervised classification: based on previous studies^17, 18^, old forest (wood volume over 100 m^3^/ha, circa 50 years or older) was classified as habitat for treecreepers, while the matrix class contained all other land classes (e.g. built-up areas, water, fields and younger forest classes). Processing and classification of satellite images is explained in more detail in Supplementary material 1.

Habitat cover was calculated at the territory core and landscape scales to study potential scale-dependent differences of the effects of change in habitat cover. A circular area extending 100 m from the nest box pair, covering an area of about 3ha, was used to describe the territory surrounding the nest box site. This was considered as the smallest possible area from which habitat area could be quantified, considering the pixel size of 30 m × 30 m of the original satellite images. Although this was smaller than the radius of 200 m used in previous studies^19, 20^ there is evidence that increased habitat cover in the immediate vicinity of the nest box site has a positive influence on a key parameter of habitat suitability, the probability of nest site occupancy^17^. In addition, this coincides roughly with the distance of least 70 m that male treecreepers defend around the nest^33^. A circular area extending 600 m, covering about 113ha, represented the landscape scale. The landscape scale was also included since the cover of habitat at this spatial scale has been shown to have both negative and positive impacts on nest predation probability in our study system^19, 20^. Pixels that had at least 50% overlap with the circular buffers representing both spatial scales were used for assessing forest cover around the nest box site. For each nest box site and year, the cover of old forest was calculated for both scales using Fragstats 3.4^34^. To avoid inconsistencies in habitat data between clouded and cloud-free territories, the percentage cover of habitat was used instead of absolute area. We then used a change trajectory-based approach to quantify the change in habitat cover through the study period for each nest box site separately. We also quantified the initial level of habitat cover for each individual nest box site, to explore whether the initial level of habitat cover at the start of the habitat series had a moderating effect on the effect of change in habitat cover.

### Statistical methods

The direct and moderated influence of initial cover and change in habitat cover on the cumulative number of fledglings of treecreepers was quantified using latent growth curve model in a structural equation modeling (SEM) framework^21, 35^. Latent growth curve modelling is an application of SEM to longitudinal data analysis, designed to partition variation of potentially multiple processes into within- and between-subject variation^36^ and, thus, it shares many characteristics with multilevel modeling^21^. Latent growth curve models used here describe initial habitat cover, rate of change over time and their covariance as latent (unobserved) intercept and slope factors with random coefficients^36^. Here, the intercept factor represents the initial cover of habitat at the beginning of the study period and the slope factor represents the change in habitat cover during the study period. The means of these parameters describe the average temporal habitat cover metrics between nest box sites (i.e. correspond to fixed parameters), while their variances describe the between-nest box site variation in these metrics (i.e. correspond to random parameters). In latent growth curve models, the paths (i.e. loadings) from latent intercept factor to the observed outcomes are fixed to 1 while the loadings of latent slope factor (i.e. time scores) represent the measurement of time since the measurement of initial habitat cover and they determine the form and centring (baseline) point of change trajectory^36^. The form of rate of change in latent growth curve modelling can take many shapes (e.g. linear, quadratic or exponential)^36^. Here, we allowed the time scores (except the first and the last time scores that were used to set the trajectory scale to obtain a change per year over our study period) to be freely estimated from the data (i.e. we used basis functions), because preliminary inspection of the data suggested nonlinear change of no clear parametric shape^36^ (Fig. 2).

In latent growth curve modelling it is possible to examine associations between multiple longitudinal processes in the same model^36^. However, owing to our modest number of nest box sites (*n* = 213), the inclusion of habitat change at both scales in addition of the longitudinal change in annual treecreeper fledgling numbers during the study period would have resulted in a grossly overfitted model (47 estimated parameters at minimum, depending on the model complexity). Therefore, the influence of change in habitat cover on the fledgling numbers of treecreepers was analysed separately for the territory core and landscape scales and by using the sum of the number of fledglings over the study years (i.e., the cumulative number of fledglings was modelled as a distal outcome). The proportion of habitat cover at both scales were treated as continuous response variables and, to aid model convergence due to too small or too large factor variances, habitat cover values were divided by 10 prior to the analyses.

Since the cumulative number of fledglings per nesting site at the end of the study period was a count variable, we started by selecting the appropriate working error distribution for this variable. This was accomplished by using sample-size adjusted Bayesian information criterion (SABIC), which is shown to perform well in model selection tasks in a SEM framework^37, 38^. Owing to the predominance of zeroes in the cumulative number of fledglings (Supplementary Fig. S2), we contrasted the following potential error distributions: Poisson and negative binomial distributions as well as their zero-inflated counterparts that consider a mixture of response distributions^39^. At both scales, zero-inflated negative binomial distribution clearly fitted the data best (Supplementary Table S2.1). This meant that we now had two regressions to estimate with respect to fledgling number: one that models how habitat cover attributes predict zero or higher number of fledglings (i.e. the count part of the mixture) and another on how the same habitat attributes predict zero fledglings only (i.e. the zero-inflation part of the mixture using logistic regression). Next, using the same model selection approach, we compared different residual variance structures of annual habitat cover because a miss-specified residual structure can bias the variance estimate of intercept and slope factors, as well as their covariance^40^. This was done by comparing models assuming the following residual variance structures in the following order: homogeneous residuals, heterogeneous residuals, homogeneous residuals with covariances with adjacent time points and heterogeneous residuals with covariances with adjacent time points. Covariances among variances between adjacent time points control for potential temporal autocorrelation between adjacent time points not accounted by the intercept and slope factors^21^. For the territory core scale, the best model included homogenous errors and estimated their residual covariances. For the landscape level, the best model fit was obtained by estimating heterogeneous residuals and estimating the residual covariances between adjacent annual habitat cover measurements (Supplementary Table S2.2).

We used a robust maximum likelihood (MLR) estimator where missing data assumed to be missing at random was handled using full information maximum likelihood estimation^41^ using Mplus 7.3^42^. In full information maximum likelihood estimation, models are estimated by giving the observations having more data points more weight compared to observations having less data points. This approach allows missing values for dependent variables, meaning here both the records of annual habitat covers as well as the numbers of fledglings. The influence of the interaction between initial habitat cover and change in habitat cover on the number of fledglings was estimated using the latent moderated structural equations method^43^. No commonly used tests and absolute fit indexes are available to assess model fit to the data, because the estimation of the current model requires the usage of individual data and hence variable(s) means, variances and covariances are not sufficient for model estimation. We tested the significance of variance components, as well as their covariance, for the slope (change in habitat cover) and intercept (initial habitat cover) using likelihood ratio tests^27^. We were not able to test for spatial autocorrelation since SEM does not produce traditional residuals from which spatial autocorrelation can be tested for. However, the results of our previous study^20^ showed that the residuals from models inspecting the influence of habitat fragmentation on the number of fledglings did not display spatial autocorrelation. Therefore, even though there was some overlap between nest box sites at the landscape (600 m) scale pseudoreplication should not be an issue here.

## Electronic supplementary material

Supplementary material
