# Supplementary material for: Using change trajectories to study the impacts of multi-annual habitat loss on fledgling production in an old forest specialist bird
Source: Sci Rep. 2017 May 12;7:1874. doi: 10.1038/s41598-017-02072-w (PMC5431986; doi:10.1038/s41598-017-02072-w)
Supplement: Supplementary file 1 — Supplementary material [file 41598_2017_2072_MOESM1_ESM.pdf]

Supplementary material

Using change trajectories to study the impacts of multi-annual habitat loss on fledgling production in an old forest specialist bird

Eric Le Tortorec[1,2*], Niina Käyhkö[3], Harri Hakkarainen[2†], Petri Suorsa[2], Esa Huhta[4], Samuli Helle[2]

[1]Department of Biological and Environmental Science, University of Jyväskylä, P.O. Box 35, FI-40014 Jyväskylä, Finland
[2]Department of Biology, University of Turku, FI-20014 Turku, Finland
[3]Department of Geography and Geology, University of Turku, FI-20014 Turku, Finland
[4]Natural Resources Institute Finland, Rovaniemi Research Unit, P.O. Box 16, FI-96301 Rovaniemi, Finland

* corresponding author
E-mail: eric.letortorec@jyu.fi

†Deceased

Supplementary material 1

Generation of landscape data

Landsat 5 Thematic Mapper satellite images containing under 10% cloud cover from six different years were downloaded from the United States Geological Survey Global Visualization Viewer service (http://glovis.usgs.gov). These images were acquired on the following dates: 31.7.1999 (path 189, row 16), 27.6.2001 (path 188, row 16), 6.6.2002 (path 188, row 16), 19.8.2003 (path 189, row 16), 2.9.2005 (path 188, row 16), 17.6.2006 (path 188, row 16) and 3.7.2006 (path 188, row 16). Due to the fact that from 2006 we had two satellite images taken 16 days apart, the cloudless areas from each image were joined in order to form a cloudless composite image. 2000 and 2004 were not represented in the satellite image time series due to high cloud cover.

Clouded areas were manually digitized and clipped out of each scene, and each image was classified into two classes: old forest as habitat for treecreepers and matrix as non-habitat. Classification was based on supervised classification using the maximum-likelihood rule using ERDAS IMAGINE 11.0. In supervised classification, the computer automatically assigns each pixel in the satellite image into a user-defined class using information from training areas, which are areas judged to be representative of a certain class, and delineated by the user. A quantitative comparison of our classified images with those produced by the Finnish Forest Research Institute showed that the old forest class corresponded to forest over 100 m3/ha of wood, which indicates forest age of over 50 years[1]. Different types of matrix class (e.g. young forests and saplings) were not classified since they could not reliably be separated from each other in the satellite images used here. The spectral separation of the two classes was good, with each separate

classification having a transformed divergence value of 2000, indicating that the classes can be spectrally separated from each other[2].

Due to the limited availability of reference data only the classification of the 2006 image could be assessed. High-resolution aerial images (0.5m/pixel) covering parts of the study site taken in May 2006 were used as a comparison. Based on a total of 10000 random pixels the constructed error matrix[3] showed that 81.1% of the sampled pixels from the classified satellite image had the same class as the corresponding pixels in the classified aerial images. The kappa coefficient[3] of 0.58 showed that the classification performed 58% better than a random classification scheme. This equates to a moderate strength of agreement, close to the limit of 0.6, which would indicate substantial agreement[4].

References

1. Tomppo, E. *et al.* Forest resources and their development in central Finland, 1967–1996. *Metsätieteen Aikakauskirja Folia For.* **2B,** 309–388 (1999).
2. Jensen, J. R. *Introductory digital image processing: a remote sensing perspective*. (Prentice Hall, 2005).
3. Lillesand, T. M., Kiefer, R. W. & Chipman, J. W. *Remote sensing and image interpretation*. (John Wiley & Sons, 2008).
4. Landis, J. R. & Koch, G. G. The Measurement of Observer Agreement for Categorical Data. *Biometrics* **33,** 159 (1977).

Supplementary material 2

Model specification

The simple linear latent growth curve model can be described by using the following equations[1].

$$y_{it} = \eta 0_i + \eta 1_i \lambda_t + \varepsilon_{it} \quad (1.1)$$

$$\eta 0_i = \eta 0 + \varsigma 0_i \quad (1.2)$$

$$\eta 1_i = \eta 1 + \varsigma 1_i \quad (1.3)$$

where equation (1.1) represents the within subject model and equations (1.2) and (1.3) represent between subject models. In equation (1.1) y_{it} is the *i*th observed outcome at time point *t*, $\eta 0_i$ and $\eta 1_i$ are random latent intercepts and slopes (i.e. growth factors), respectively, and λ_ts are time scores for the growth factors. The residual ε_{it} in equation (1.1) includes influences of both random measurement error and time-specific influence of the *i*th individual at time *t*. In between subject models (equations (1.2) and (1.3)), the random latent intercept and slopes, $\eta 0_i$ and $\eta 1_i$, respectively, are modelled as a function of their respective overall means, $\eta 0$ and $\eta 1$, respectively, as well as error terms ($\varsigma 0_i$ and $\varsigma 1_i$) representing between subject variation in regard to the *i*th individual growth trajectory.

Model selection procedure

We applied two sequential model selection episodes, based on sample-size adjusted Bayesian information criteria (SABIC), to obtain a final model separately for both spatial scales. We started by searching for the best fitting model to account for the error distribution for the number of fledglings (Figure S2). Alternatives considered were Poisson and negative binomial distributions as well as their zero-inflated counterparts (Table S2.1). After this, we contrasted the fit of latent growth curve models assuming homogeneous residuals, heterogeneous residuals, homogeneous residuals with covariances between adjacent time points and heterogeneous residuals with covariances between adjacent time points (Table S2.2). At the territory core scale, the model having heterogeneous residuals with covariances between adjacent time points failed to converge irrespective of several attempts to fine-tune the converge process. Therefore, the model with homogenous residual variances and pairwise residual covariances was chosen as the best-fitting model for the territory core scale.

Table S2.1. Model selection of error distribution for the count outcome, the summed number of fledglings. The best-fitting models are marked in bold.

	100m	600m
Error distribution	SABIC	SABIC
Poisson	5855.3	3754.1
Negative binomial	5633.9	3497.1
Zero-inflated Poisson	5648.2	3633.9
Zero-inflated negative binomial	**5569.7**	**3429.2**

Table S2.2. Model selection of residual variance structure for the annual habitat cover levels. The best-fitting final models are marked in bold.

	100m		600m	
	# parameters	SABIC	# parameters	SABIC
Homogenous residual variances	19	5557.6	19	3424.812
Heterogeneous residual variances	24	5551.1	24	3376.023
Homogenous residual variances with pairwise residual covariances	**24**	**5461.9**	24	3337.057
Heterogeneous residual variances with pairwise residual covariances	no convergence		**29**	**3315.318**

Fig S2. Histogram showing the distribution of the number of fledged offspring per nest box site, summed for the study duration.

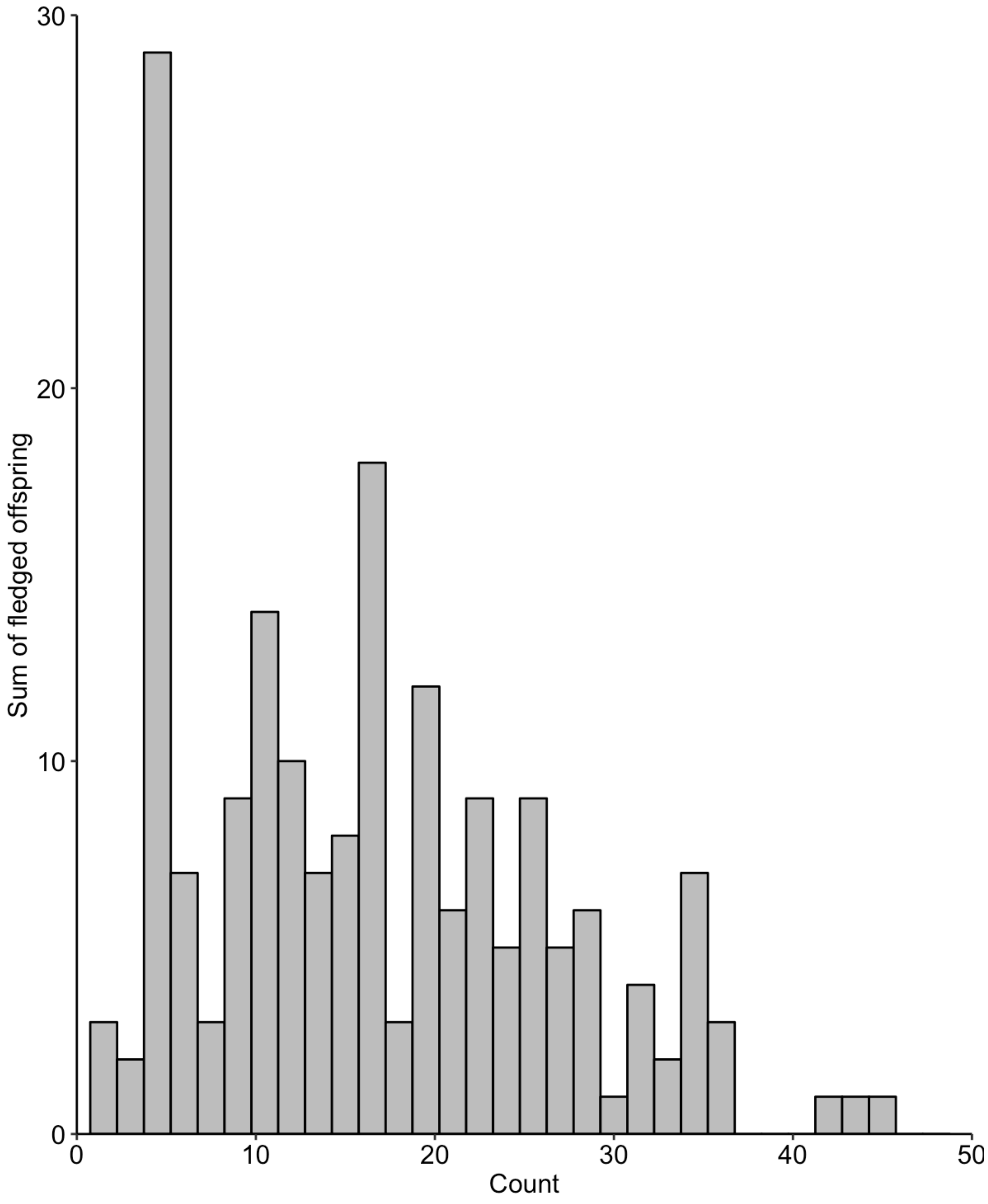

30
20
10
0
Sum of fledged offspring
0
10
20
30
40
50
Count

References

1. Bollen, K. A. & Curran, P. J. *Latent curve models: a structural equation perspective*. (Wiley-Interscience, 2006).
